# Chronic constipation in a 9-year-old male: a case of Hirschsprung disease with complex transition zone pathology

**DOI:** 10.11604/pamj.2026.54.21.49617

**Published:** 2026-05-28

**Authors:** Gahana Kataria, Vaishali Dhawan, Pratapsingh Parihar

**Affiliations:** 1 Department of Radio-Diagnosis, Jawaharlal Nehru Medical College, Datta Meghe Institute of Higher Education and Research (DU), Sawangi (Meghe), Wardha, Maharashtra, India

**Keywords:** Hirschsprung disease, chronic constipation, pediatric surgery, transition zone, case report

## Abstract

Hirschsprung disease (HD) is a congenital condition usually diagnosed in neonates, but it can present later in childhood with persistent constipation. We describe the case of a 9-year-old boy with a history of recurrent constipation since infancy, who presented with severe abdominal distension. Radiological investigations revealed a transition zone consistent with Hirschsprung disease, and histopathology showed variable hypoganglionosis. He underwent exploratory laparotomy with a double-barrel colostomy, recovering without complications. This case highlights the diagnostic challenges in older children and emphasises the importance of thorough histopathological sampling in defining transition zones. It also highlights the importance of long-term follow-up, particularly in terms of functional outcomes, for patients with Hirschsprung disease

## Introduction

Hirschsprung disease (HD) is a congenital disorder affecting approximately 1 in 5,000 live births, characterised by the absence of ganglion cells in the distal bowel, resulting in functional obstruction [1]. While typically diagnosed in the neonatal period with delayed meconium passage, approximately 10-15% of cases present later in childhood with chronic constipation [1,2]. The diagnosis relies on showing aganglionosis through rectal biopsy, though recent advances in immunohistochemistry, particularly calretinin staining, have improved diagnostic accuracy [3].

## Patient and observation

**Patient information:** a 9-year-old male presented to the emergency department at 3 am with a 4-day history of constipation and abdominal distension. The mother reported that the child had been experiencing recurrent episodes of constipation for several years, with multiple hospitalisations requiring symptomatic management. The current episode was characterised by complete cessation of bowel movements with only passage of flatus at night. The patient's past medical history included a birth weight of 2.5 kg via normal vaginal delivery, followed by NICU admission for one month due to respiratory distress syndrome. He had experienced multiple episodes of constipation since early childhood that were treated symptomatically at various hospitals. A previous abdominal ultrasound performed in May had shown distended bowel loops. There was no history of blood in stools, vomiting, or seizures. His developmental milestones were normal, and he was completely immunised according to schedule. The parents had a non-consanguineous marriage.

**Clinical findings:** on examination, vital signs were as follows: heart rate 74 beats per minute, respiratory rate 25 breaths per minute, blood pressure 100/72 mmHg, and oxygen saturation 99% on room air. The abdomen was soft but significantly distended with an everted umbilicus and was non-tender to palpation. Cardiovascular examination showed normal S1 and S2 heart sounds without murmurs. Respiratory examination showed clear breath sounds bilaterally. The child was alert and conscious with no neurological deficits.

**Diagnostic assessment:** radiological findings included plain abdominal radiographs showing multiple dilated bowel loops with air-fluid levels suggesting distal pseudo-obstruction (Figure 1A-F). Contrast enema examination was carried out using 700-800 ml of diluted contrast, revealing gross dilatation from the rectum to the descending colon, with specific measurements: rectum 12.5 cm, sigmoid colon 7.8 cm, and descending/transverse colon 4.5 cm. The study demonstrated a relatively narrow distal bowel segment with marked proximal colonic dilatation, revealing a transition zone characteristic of Hirschsprung disease. The massively dilated proximal colon showed extensive faecal loading, with poor contrast material evacuation. Contrast-enhanced CT abdomen showed grossly dilated proximal rectum, sigmoid colon, and distal descending colon with maximum calibre measuring 9 cm (loaded with faecal matter), while the distal rectum remained non-dilated (Figure 2A-C). The transverse colon appeared collapsed, and the ascending colon was of normal calibre. The radiologist's impression specifically noted that findings were suspicious of Hirschsprung disease and recommended rectal biopsy. Follow-up ultrasound showed dilated sigmoid and large bowel loops with partial visualisation of abdominal organs due to reverberation artefacts; however, peristalsis was noted in visualised loops of the left lumbar and iliac regions. Histopathological examination of multiple specimens obtained during surgical intervention showed variable findings. Container 1, containing tissue from the peritoneal reflection, consisted of a single greyish-white tissue measuring less than 0.5 x 0.5 cm that was negative for hypoganglionosis on histopathology (Figure 3A-D). Container 2, containing rectal tissue, showed a single greyish-white tissue measuring 0.5 x 0.5 cm that was also negative for hypoganglionosis. Container 3, from the rectosigmoid region, contained a single irregular greyish-white tissue measuring 0.5 x 0.5 cm that showed hypoganglionosis on histopathological examination. Container 4, from the descending colon, contained two irregular greyish-white tissue pieces aggregating 0.5 x 0.5 cm that showed hypoganglionosis (Figure 4A-C). Container 5, from the distal stoma site, contained a single irregular greyish-white tissue measuring 0.5 x 0.5 cm that also showed hypoganglionosis on histopathology.

**Figure 1:**
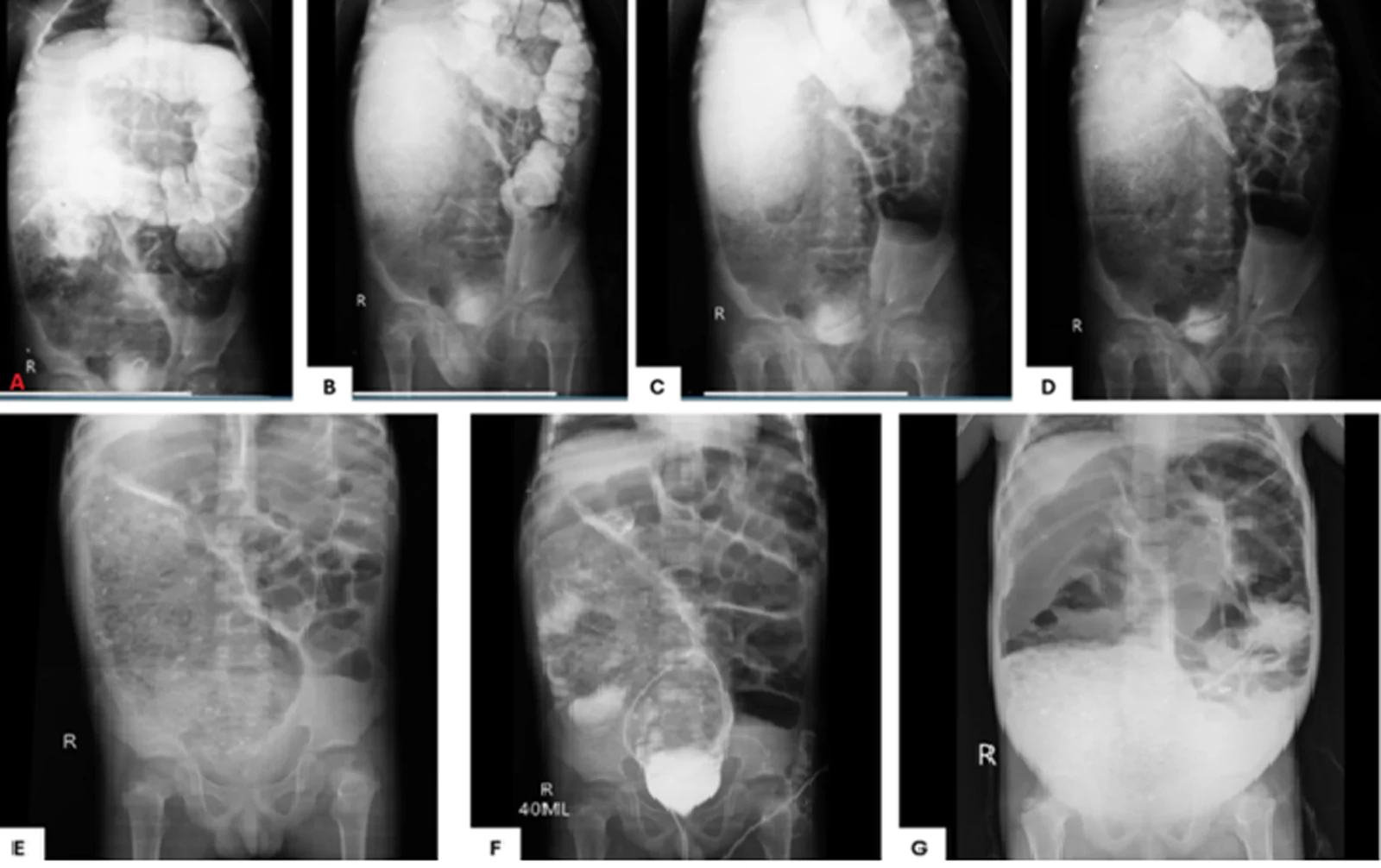
A-F) serial contrast enema images demonstrating a transitional zone with proximal colonic dilatation suggestive of Hirschsprung disease; post-contrast abdominal radiograph shows persistent bowel distension and faecal loading

**Figure 2:**
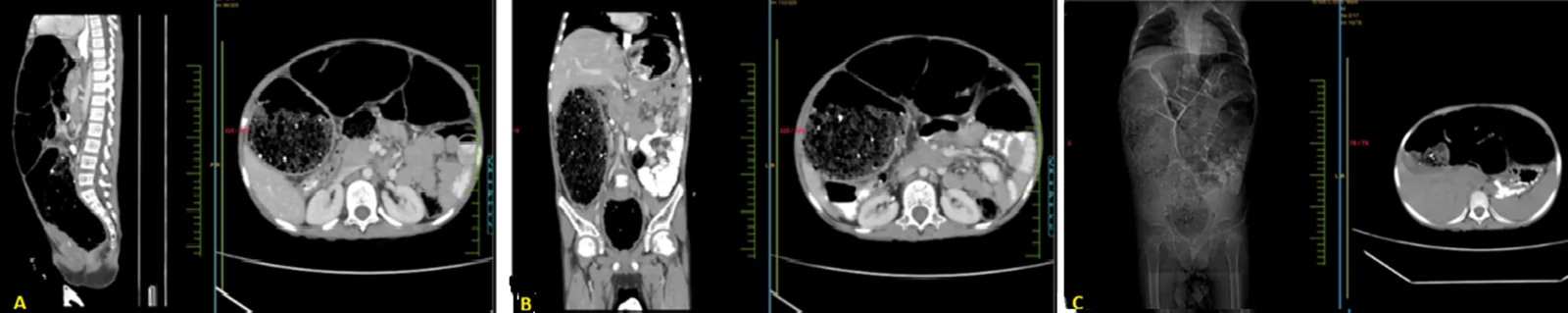
contrast-enhanced CT abdomen and pelvis in; A) sagittal and axial; B) coronal; C) scout CT images showing markedly dilated proximal rectosigmoid colon with non-dilated distal rectum suggestive of imaging findings in Hirschsprung disease with distal pseudo-obstruction

**Figure 3:**
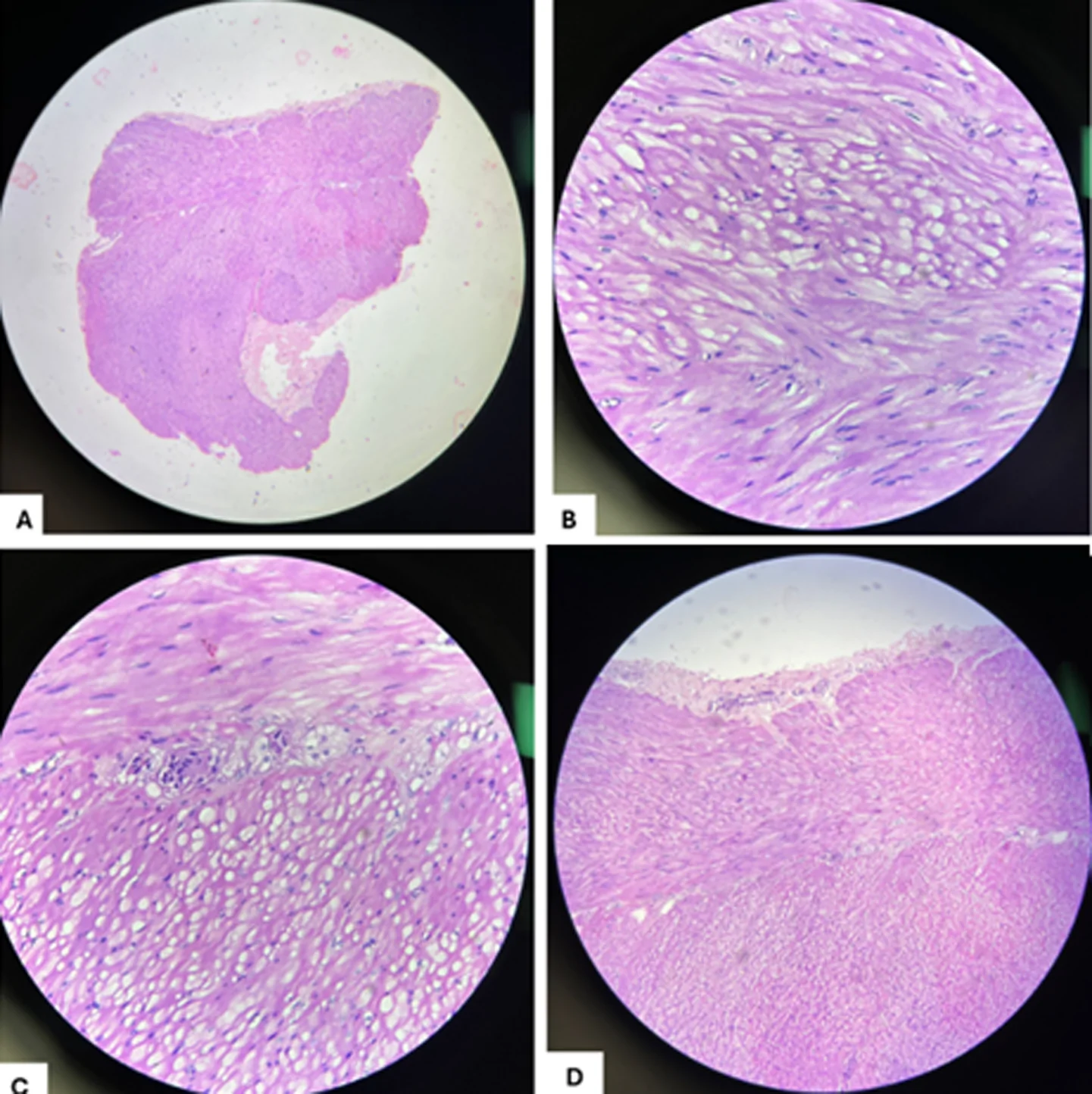
ganglionic bowel showing normal myenteric ganglion cells: A) low-power view of ganglionic bowel showing preserved architecture; B-D) high-magnification of myenteric plexus with visible ganglion cells between muscle layers showing normal ganglion cell density; D) H&E stain; A) x40; B-D) x100

**Figure 4:**
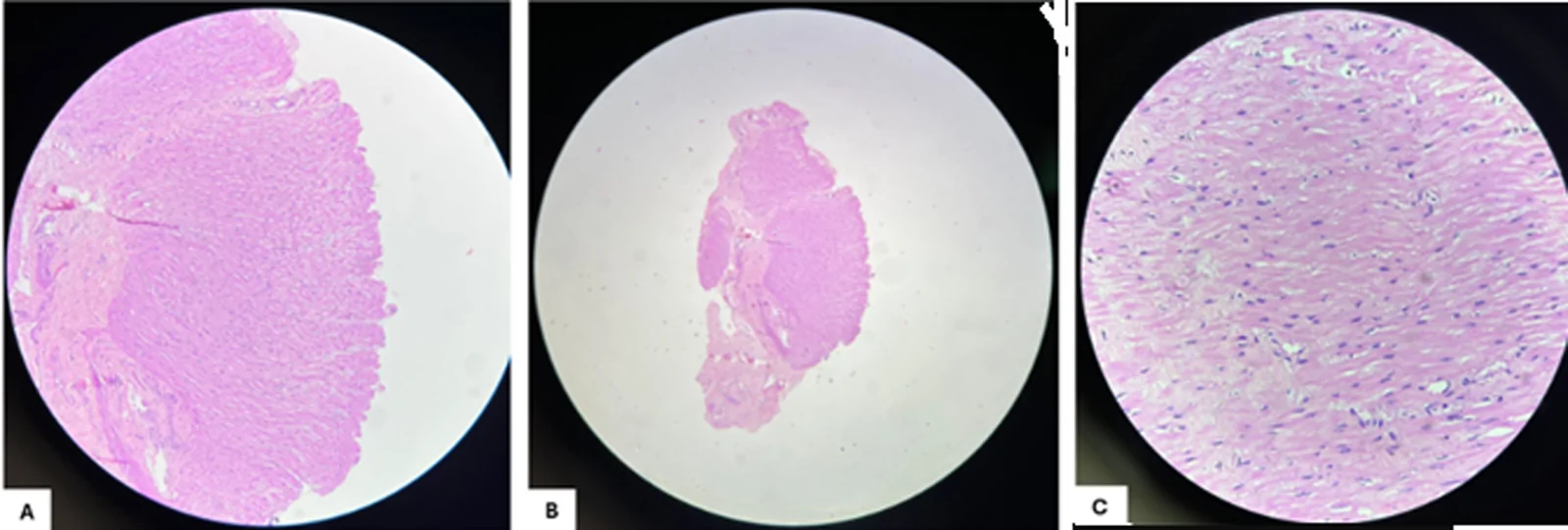
hypoganglionic bowel segments showing reduced myenteric ganglion cells; A, B) low-power views of hypoganglionic bowel segments showing preserved overall architecture; C) high-magnification of myenteric plexus region showing marked hypoganglionosis with severely reduced ganglion cell density; H&E stain; A, B) x40; C) x100

**Therapeutic intervention:** based on clinical presentation and investigation findings, the patient had an exploratory laparotomy with creation of a colostomy with a double-barrel loop stoma. The procedure was carried out under general anaesthesia without complications. Postoperative recovery was without complications.

**Timeline:** the child had a history of recurrent constipation since infancy, for which he underwent multiple hospital visits and received only symptomatic treatment, providing temporary relief. In May 2023, an abdominal ultrasound revealed dilated bowel loops, and the patient was managed conservatively at that time. Four days before the current hospital admission, at the age of nine years, he developed severe constipation and abdominal distension, passing only minimal flatus. He was admitted to the emergency department for evaluation. On the day of admission, clinical assessment and imaging studies, including plain abdominal radiographs, contrast enema, and CT scan, showed dilated proximal colon with a transition zone suggestive of Hirschsprung disease, leading to surgical consultation. During the same admission, the child underwent exploratory laparotomy with the creation of a double-barrel colostomy. Multiple biopsies were taken, which showed mixed histopathological findings of hypoganglionosis and normal tissue.

**Follow-up and outcomes:** the surgery was uneventful, and postoperative recovery in the ward was smooth, with early resumption of oral intake and good stoma function. He was discharged in stable condition. During short-term follow-up, the child remained asymptomatic with no complications, and a definitive pull-through surgery has been planned for a later date.

**Patient perspective:** the child´s mother described a long history of constipation that often led to repeated hospital visits and significant stress for the family. She expressed relief that a clear diagnosis was eventually established and that surgery brought immediate improvement. The family is optimistic that the planned definitive surgery will allow the child to live more comfortably without recurrent hospitalisations.

**Informed consent:** written informed consent was obtained from the patient´s parents for publication of this case report, including the use of clinical details and images. A copy of the signed consent form is available for review by the editorial office of the journal.

## Discussion

This case illustrates several important aspects of HD diagnosis and management in older children, particularly highlighting the complexity of transition zone pathology and its implications for surgical planning. Our patient's presentation at 9 years of age with chronic constipation illustrates the subset of HD cases that escape early diagnosis. While 90% of HD cases present with delayed meconium passage in the neonatal period [1], approximately 10-15% present later with chronic constipation [1,2]. The diagnostic workup in our case showed variable histopathological findings, with some specimens showing hypoganglionosis while others were negative. This pattern aligns with Kapur *et al*. detailed characterisation of transition zone histopathology, which identified six distinct features, including myenteric hypoganglionosis, with the transition zone length varying widely from 0-12 cm depending on which feature defines its proximal boundary [4]. Their work emphasised that transition zone abnormalities show variable proximal extension, explaining the mixed histopathological results in our case. The variable histopathological findings across different bowel segments mirror findings from Kapur *et al*. systematic analysis of transition zones [4]. The presence of hypoganglionosis in some specimens but not others suggests sampling from different points along the transition zone continuum. This variability underscores the importance of the ERNICA guidelines' recommendation to resect 5-10 cm of normally ganglionic bowel beyond the apparent transition zone to ensure complete removal of abnormal tissue [5]. The imaging findings in our case, specifically the contrast enema showing a transition zone with rectum measuring 12.5 cm, sigmoid 7.8 cm, and descending/transverse colon 4.5 cm in diameter, along with CT findings showing maximum colonic caliber of 9 cm with non-dilated distal rectum, are consistent with distal obstruction and were highly suggestive of Hirschsprung disease, prompting the radiologist to specifically recommend rectal biopsy.

Recent advances have established calretinin immunohistochemistry as superior to traditional acetylcholinesterase staining, with a sensitivity of 100% and specificity of 99.2% [3]. The presence of hypoganglionosis rather than complete aganglionosis in our patient illustrates a variant form that can complicate diagnosis and management. Ambartsumyan *et al*. emphasised that adequate tissue sampling is crucial, with 30% of false-negative biopsies attributed to inadequate depth [6]. Our case used multiple-level biopsies, revealing the patchy nature of ganglionic abnormalities. This approach aligns with ERNICA guidelines recommending comprehensive tissue sampling for accurate diagnosis [5]. The decision to perform colostomy with a double-barrel loop stoma in our patient indicates appropriate management given the diagnostic uncertainty and recurrent symptoms. While current practice favours single-stage pull-through procedures when feasible, staged procedures remain indicated for patients with enterocolitis, diagnostic uncertainty, or significant bowel distension. Georgeson *et al*. pioneering work on primary laparoscopic pull-through [7] and De la Torre-Mondragón *et al*. transanal approach [8] have revolutionised HD surgery, but these techniques require a clear definition of the transition zone. Our patient's variable histopathology necessitated a more conservative staged approach to ensure complete resection of abnormal bowel while preserving continence mechanisms. Long-term studies provide sobering context for our patient's prognosis. Dai *et al*. meta-analysis showed persistent faecal incontinence in 20% of patients over age 10 and ongoing constipation in 14% [9]. Long-term functional problems affect a significant proportion of HD patients, with constipation persisting in many cases [10]. These findings emphasise the need for comprehensive long-term follow-up and management strategies. Risk factors for poor outcomes identified in these studies include total colonic aganglionosis, Down syndrome, and postoperative enterocolitis. While our patient lacks these specific risk factors, the late presentation and transition zone complexity may impact long-term functional results.

Given our patient's history of distended bowel loops and multiple constipation episodes, Hirschsprung-associated enterocolitis (HAEC) prevention is crucial. Our patient requires careful monitoring for HAEC symptoms, including fever, abdominal distension, and explosive diarrhoea, with prompt treatment with bowel rest, metronidazole, and rectal irrigations if symptoms develop. Although genetic testing was not performed in our case, current understanding of HD genetics warrants discussion. The comprehensive review by Montalva *et al*. identified over 20 genes involved in HD pathogenesis, with RET mutations accounting for 50% of familial cases [1]. Genetic counselling should be offered to the family, particularly given the implications for future pregnancies with a recurrence risk of 3-4% for short-segment disease. Planning for definitive pull-through surgery requires careful consideration of the proximal extent of disease. The ERNICA guidelines recommend frozen section evaluation during definitive surgery to confirm normally ganglionic bowel at the anastomotic site [5]. Given our patient's age and the successful diversion, the timing of the pull-through can be individualised based on growth, nutritional status, and bowel preparation.

## Conclusion

This case highlights the diagnostic complexity of Hirschsprung disease presenting with chronic constipation in older children. The variable histopathological findings emphasise the importance of comprehensive tissue sampling and careful evaluation of transition zone pathology. While advances in surgical techniques have improved outcomes, accurate diagnosis and complete resection of abnormal bowel remain fundamental to successful management. Long-term follow-up with attention to functional outcomes and HAEC prevention will be essential for optimising this patient's quality of life.
